# Investigation of the molecular characteristics of *Brucella* isolates from Guangxi Province, China

**DOI:** 10.1186/s12866-019-1665-6

**Published:** 2019-12-16

**Authors:** Zhi-guo Liu, Miao Wang, Hong-yan Zhao, Dong-ri Piao, Hai Jiang, Zhen-jun Li

**Affiliations:** 1 State Key Laboratory for Infectious Diseases Prevention and Control, National Institute for Communicable Disease Control and Prevention, Chinese Center for Disease Control and Prevention, 155 Changbai Road, Changping, Beijing, 102206 People’s Republic of China; 2 Inner Mongolia Autonomous Region Comprehensive Center for Disease Control and Prevention, Huhhot, 010031 People’s Republic of China; 3 Ulanqab Centre for Endemic Disease Prevention and Control, Jining, 012000 Inner Mongolia China

**Keywords:** *B. melitensis*, *B. suis*, MLVA, MLST, Molecular characteristics, Guangxi

## Abstract

**Background:**

Human brucellosis has become a severe public health problem in China’s Guangxi Province, and there has been higher prevalence of brucellosis in this region after 2010. Both multiple locus variable-number tandem repeat analysis (MLVA) and multilocus sequence typing (MLST) assay schedules were used to genotype isolates and determine relationships among isolates.

**Results:**

A total of 40 isolates of *Brucella* were obtained from humans, pigs, and dogs from 1961 to 2016. There were at least three species of *Brucella* detected in Guangxi Province, *Brucella melitensis*, *Brucella suis*, and *Brucella canis*, with 16, 17, and 7 isolates, respectively. Of which *B. suis* biovar 3 was the predominant species resulting in pig brucellosis in the area examined before 2000s. Moreover, *B. melitensis* biovar 3 was found to be mainly responsible for human brucellosis during 2012–2016. All *B. melitensis* isolates in this study belonged to East Mediterranean lineage. MLVA-11 genotype 116 was the dominant genotype and represented 81.2% of the isolates. MLVA cluster analysis showed there to be 44% (7/16) brucellosis cases caused by *B. melitensis* with a profile of outbreak epidemic from 2012 to 2016. However, nearly 83.3% (20/24) of brucellosis cases resulting from both *B. suis* and *B. canis* showed no epidemiological links or sporadic characteristics. MLVA-16 analysis confirmed extensive genotype-sharing events between *B. melitensis* isolates from Guangxi and other northern provinces within China. These data revealed that there are potential epidemiology links among these strains. *B. suis* strains of this study showed a unique genetic lineage at the global level and may have existed historically in this area. However, present *B. canis* isolates were closely related to previously reported isolates in Korea, where they may have originated. MLST typing showed that the population structure of *Brucella* strains had changed considerably in this province; ST17 and ST21, two previously predominant populations appeared to have been replaced by recently emerging ST8 group.

**Conclusions:**

Our investigation data have inspired the hypothesis that Guangxi Province had been subject to an imported human brucellosis epidemic. Our data suggest that strains found in Northern regions of China are the principal source of infections in recent cases of human brucellosis in Guangxi Province. Comparative genomic analysis from more strains is necessary to confirm this hypothesis. This work will facilitate better understanding of the epidemiology and improve the effectiveness of control and prevention of brucellosis in this region.

## Background

Brucellosis is a common zoonotic disease caused by the genus *Brucella*, a Gram-negative, facultative intracellular bacteria that infects a wide range of mammals, including domestic and wild animals as well as humans [1, 2]. Human brucellosis is largely dependent on the animal reservoirs and spreads through direct contact with infected animals or consumption of contaminated animal products [3]. *B. melitensis, B. abortus*, and *B. suis* are highly pathogenic and a frequent causative pathogen of animal and human brucellosis [4]. *B. melitensis* is the predominant circulating strain in northeastern and northwestern China and it produces the most severe infections in humans [5]. *B. abortus* is the main etiological agent of brucellosis in cattle, but it is associated with far fewer cases of human disease [6, 7]. Human cases due to *B. abortus* are sporadic in China and, in Sichuan, *B. abortus* is the predominant strain isolated. It generally causes infections less severe than those caused by *B. melitensis* or *B. suis* [8, 9]. *B. suis* is the most disseminated species in south regions of China and are associated with sporadic brucellosis epidemics [10]. Recently, due to unrestricted movements of a large number of infected small ruminants and other illegal trade, the geographic distribution profiles of *Brucella* species and transmission patterns have changed [11]. The identification and molecular typing of the circulate *Brucella* species is extremely essential for epidemiological follow-up and control of the disease [12]. Classical biotyping is the gold standard for investigations of phenotypic characteristics, and deeper molecular epidemiological investigations can be used to trace back the source of infection to its geographic origin and determine relationships among isolates [13].

Guangxi Province was the site of an historic brucellosis epidemic; *B. suis* predominates in this region [9]. The first case of brucellosis was reported in 1952 and found to have been caused by imported infected pigs from other regions. Some *B. suis* strains were later isolated [14]. Human brucellosis is limited to few cases every year and its incidence was low before 1990. Most of these cases of brucellosis were caused by *B. suis* and *B. canis*. Since 2012, the number of human brucellosis cases has increased every year [15]. However, molecular typing and epidemiology characteristics of *Brucella* isolates from Guangxi remain unknown. In this report, we present the results of a study performed by *Brucella* MLVA16 assay on 40 human and animal field isolates of *B. melitensis*, *B. suis*, and *B. canis*. The aims of this study were to investigate causes of the increase in the incidence of human brucellosis and to estimate the epidemiological relationship of isolates for a trace-back survey of the source of infections in Guangxi, China.

## Methods

### Bacterial strains and DNA preparation

A total of 40 isolates were isolated from Guangxi province between 1961 and 2016 year. They represent all *Brucella* isolates collected during this period in Guangxi province. These strains were isolated from human blood, dog and pig at the first line laboratory by the Guangxi Center for Infectious Disease Control and Prevention. 18 were recovered from human blood, 15 from pig, and 6 from dog. *B. melitensis* bv. 1 16 M was used as control strain to calibrate the VNTR units. DNA was isolated using Full-automatic nucleic acid extraction system (LLXBIO China Ltd., China) extraction from 48-h cultures according to the manufacturer’s instructions. DNA extracted from all isolates was stored at − 20 °C.

### *Brucella* biotyping

All isolates were identified as *Brucella* species on the basis of morphology and conventional identification methods according to standard biotyping procedures, including requirement of CO_2_ for growth, H_2_S production, sensitivity to thionin (10 and 20 μg/ml), basic fuchsin (20 μg/ml) and agglutination with mono-specific antiserum for A and M antigens and phage lysis test (*Tbilisi, Tb; Berkeley, Bk2; Weybridge; Wb*) [16, 17]. Reagents and pages were obtained from the National Institute for Communicable Disease Control and Prevention, which is China’s center for infectious disease control and prevention. *B. melitensis* 16 M (BM) and *B. abortus* 544 (BA) and *B. suis* 1330 (BS) were used as control strains.

### MLVA-16 genotyping

All of isolates were further examined by MLVA, genotyping schedule, PCR amplification process as described previously [13, 18]. PCR products were preliminarily evaluated by 2% or 3% agarose gel electrophoresis. Then, positive products were denatured and resolved by capillary electrophoresis on an ABI Prism 3130 automated fluorescent capillary DNA sequencer (Applied Biosystems). Fragments were sized following comparison with a ROX (carboxy-X-rhodamine)-labeled molecular ladder (MapMaker 1000; Bioventures Inc., Murfreesboro, TN, USA) and Gene Mapper software version 4.0 (Applied Biosystems). The fragment sizes were converted to repeat unit numbers using a published allele numbering system [19].

### MLST genotyping

MLST genotyping was performed by analyzing nine distinct genomic locus, including seven housekeeping genes (gap, aroA, glk, dnaK, gyrB, trpE, and cobQ), one outer membrane protein (omp25), and one intergenic fragment (int-hyp) [20]. PCR amplification was performed as described previously [21]. Sequences obtained from purified PCR products were aligned using MEGA 6.0 software according to published MLST sequences in GenBank (accession numbers AM694191-AM695630) [20]. A local comparison database was established after downloading of relevant data, and distinct alleles identified at the nine selected loci were each given a numerical designation according to the sequences of the defined alleles. Each sequence type over all loci (ST) was predicted by comparisons and analyses based on a local comparison database established using MEGA 6.0 and a web-based MLST service (*Brucella* Base, https://pubmlst.org/brucella/). DNA preparations from the *B. melitensis* 16 M, *B. abortus* 544, and *B. suis* 1330 reference strains were used as controls.

### Analysis of genotyping data

MLVA data were analyzed using BioNumerics version 7.6 software (Applied Maths, Belgium). Both categorical coefficient and un-weighted pair group methods with arithmetic mean algorithm (UPGMA) were applied to MLVA clustering analysis (Additional file 1: Table S1). Resultant genotypes were compared using the online *Brucella* 2016 MLVAbank. The MLVA-11 characters (combination of panels 1 and 2A loci) of the isolates were compared to those of strains in the 2016 MLVA bank to determine the geographic origin of each strain; minimum spanning tree (MST) based on complete MLVA-16 was used to investigation molecular relationships between strains in this study and 340 isolates including three species: *B. melitensis* (*n* = 296), *B. suis* (*n* = 15), and *B. canis* (*n* = 29) from other provinces of China (MLVAbank_V1.4.0) (Additional file 2: Table S2). MLVA-16 was used for genetic relationship investigation of both *Brucella suis* and *canis* on a global scale (*n* = 615) (Additional file 3: Table S3). The resulting MLST genotypes were compared using the web-based MLST database (https://pubmlst.org/brucella/) (Additional file 4: Table S4). MLST dendrogram was constructed by BioNumerics version 7.6 software. MLVA and MLST profiles of 40 isolates have been submitted to the MLVA bank_V1.4.0 (http://microbesgenotyping. i2bc. paris-saclay.fr/).

## Results

### Characteristics and distributions of isolates

All strains from this study exhibited a convex, circular, and translucent morphology profile. A slight blue color was observed under sunlight and gram-negative coccobacilli were observed, resembling fine sand under the microscope. The growth characteristics, phage lysis experiments, dye bacteriostatic tests, and slide agglutination with monospecific anti-*Brucella* sera were used to characterize all isolates (Table 1). Species and biovar were discriminated based on standard bacteriological procedures. Finally, biotyping identified 16 strains as *B. melitensis* biovar 3, 18 strains as *B. suis* (biovar 1 (*n* = 2) and biovar 3 (*n* = 15)), and 7 as *B. canis.* 19 strains were isolated from human blood, 21 out of 40 from animals (19 from pigs and 6 from dogs) (Table 2). Geographic distribution profiles of some strains were unknown (according to requests made by the local department of health, it is not permitted to publish the locations of *B. melitensis* isolates).

**Table 1:** Biotyping characteristics of *Brucella* species isolates in Guangxi, China

Strain No.	Growth characteristics				Monospecific Sera			Phages lysis testing			Interpreted
	CO_2_ requested	H_2_S	BF	TH	A	M	R	Tb	BK_2_	Wb	
BA	+	+	+	–	+	–	–	+	+	+	*B. abortus 544*
BM	–	–	+	+	–	+	–	–	+		*B. melitensis16M*
BS	–	++	–	+	+	–	–	–	+	+	*B. suis 1330*
GX001~015, GX040	–	–	+	+	+	+	–	NL	CL	NL	*B. melitensis bv. 3*
GX021, GX024	–	++	–	+	+	–	–	NL	CL	CL	*B. suis bv. 1*
GX016~021, 022, 023, GX025~032	–	–	+	+	+	–	–	NL	CL	CL	*B. suis bv. 3*
GX033~GX039	–	–	–	+	–	–	+	NL	NL	NL	*B. canis*

Strain No., the number conferred to isolates;

BF, Basic fuchsin at 20 μg /ml (1/50,000 w/v); TH, Thionin at 20 μg /ml (1/50,000 w/v);

Phages, Tb = Tbilisi, BK_2_ = Berkeley type 2, Wb = Weybridge;

CL, Confluent Lysis; NL, No lysis; RTD, Routine test dilution;

+, positive (serum agglutination positive);

-, negative (serum agglutination negative)

**Table 2:** Host, sample ID, and sample size distribution of *Brucella* species in Guangxi, China

Location	Host	Sample ID	Sample size
Guangxi	Human	GX001–015, GX018–020, GX040	19
	Pig	GX016, GX017, GX021–032, GX036	15
	Dog	GX033–036, GX037–039	6

Location, the regions of sample collection

Host, the hosts from which the bacteria were isolated

Sample ID: serial number for the 40 isolates

### MLVA typing of isolates

**Fig. 1:**
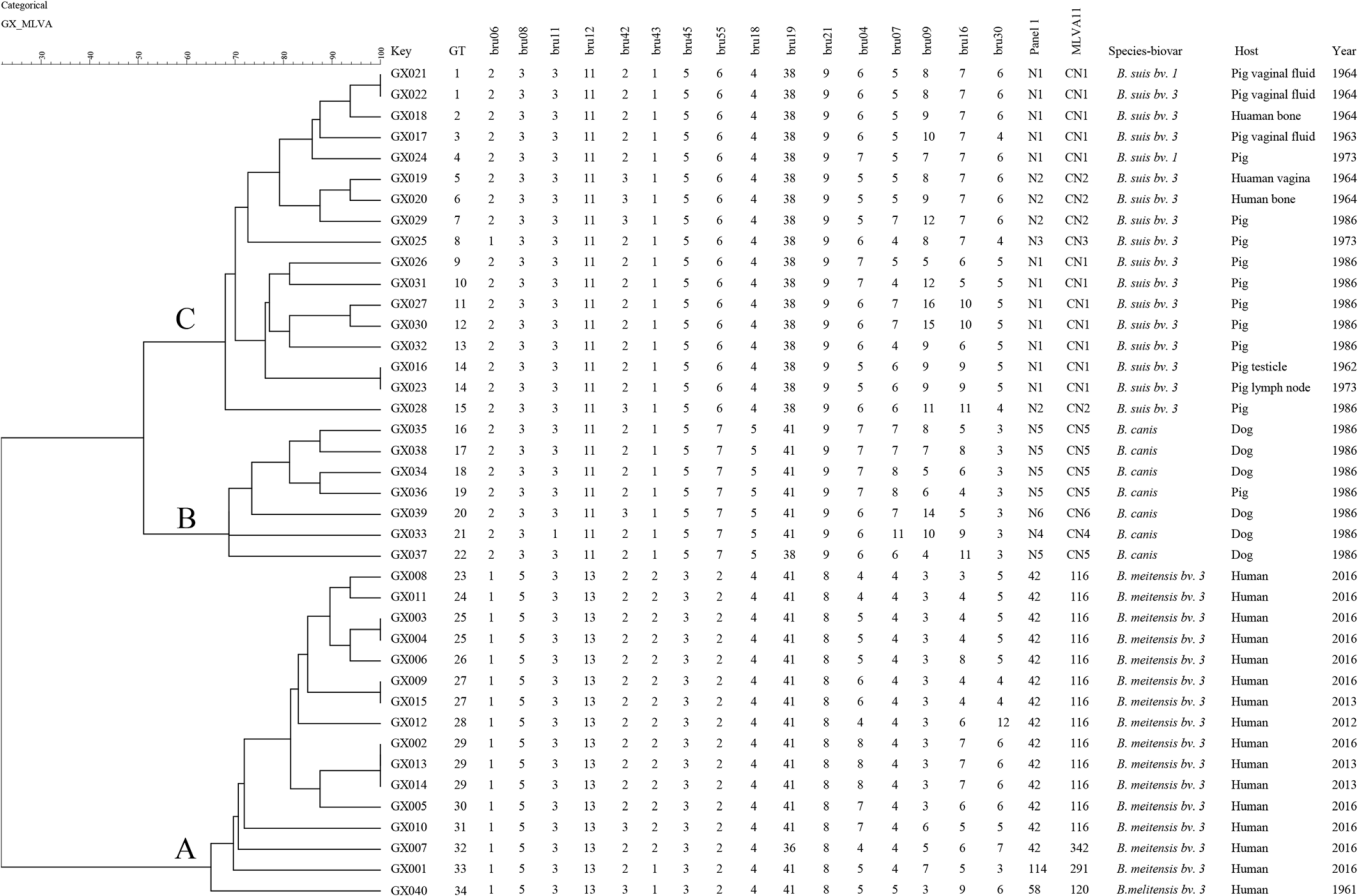
Dendrogram based on the MLVA-16 genotyping assay (UPGMA method), showing relationships between the 40 *Brucella* isolates. The columns show the identification numbers, MLVA-16 genotypes (GT), panel 1 genotypes and MLVA-11 (panels 1 and 2A) genotypes, species-biovar, host, and the year of isolation of the strains

Using panel 1 markers, the present population clustered into nine genotypes: 42 (1–5- 3-13–2-2-3-2; *N* = 14), 114 (1–5–3-13–3-2-3-2; *N* = 1), 58 (1–5–3-13–3-1-3-2; *N* = 1), N1 (2–3-3- 11 -2-1-5-6; *N* = 12), N2 (2–3–3-11–3-1-5-6; *N* = 4), N3 (1–3–3-11-2-1-5-6; *N* = 1), N4 (2–3–1-11-2- 1-5-7; *N* = 1), N5 (2–3–3-11-2-1-5-7; *N* = 5) and N6 (2–3–3-11–3-1-5-7; *N* = 1). Clustering analysis showed that the 40 isolates formed three main clusters (A–C) (Fig. 1). Cluster A had three genotypes (42, 114 and 58); cluster B had three new genotypes (N4-N6), cluster C had also three new genotypes (N1 - N3). Using the *Brucella* 2016 MLVA database and panel 1 markers, found samples contained three species: *B. melitensis* (genotypes 42, 58, and 114; cluster A), genotypes 42 represent 87.5% (14/16) of isolates and plays a dominant role in *B. melitensis*. *B. suis* (genotype N1–N3; cluster C and B) and *B. canis* (genotype N4–N6; cluster B).

### MST characterization of isolates based on MLVAs analysis

**Fig. 2:**
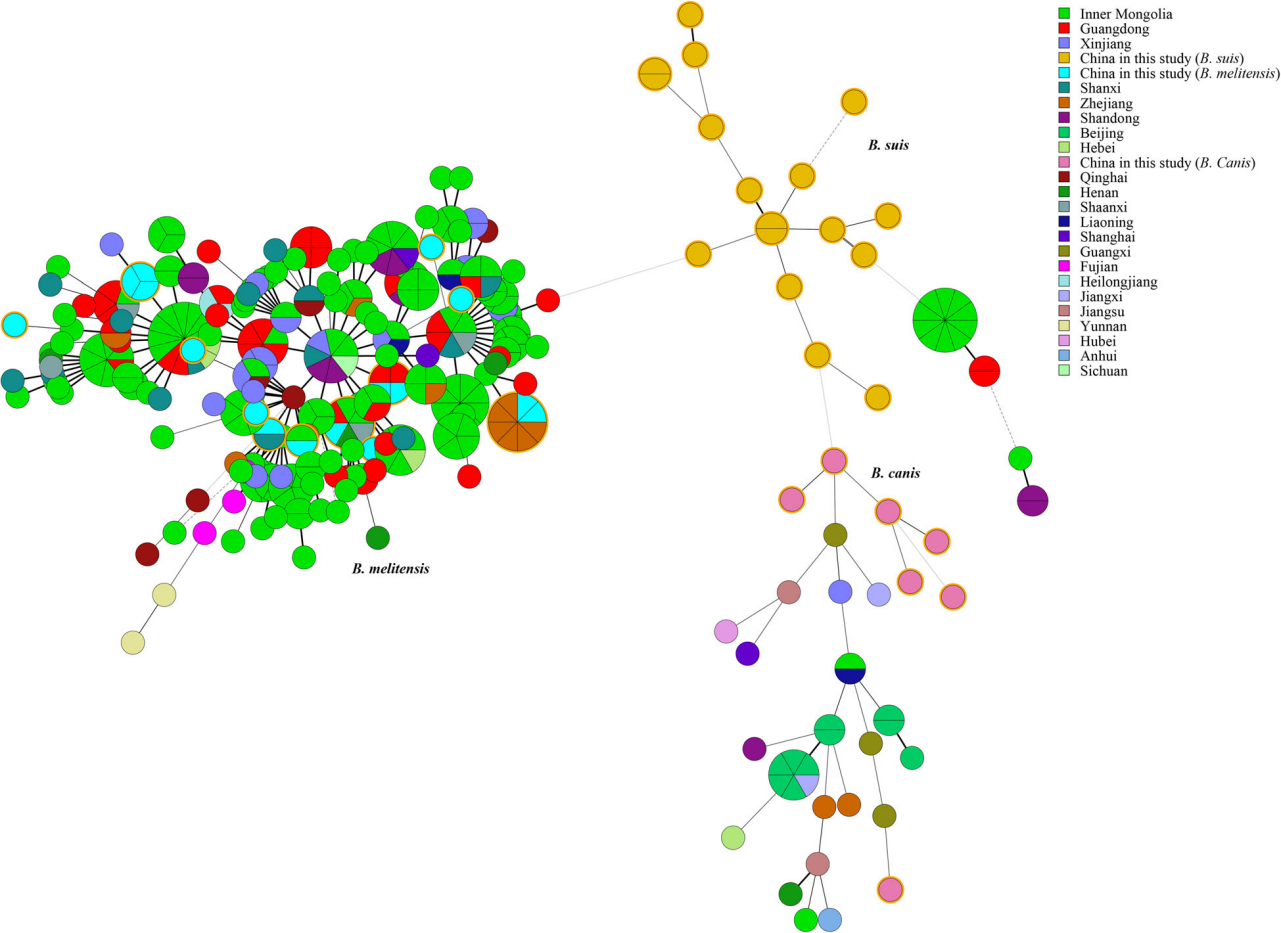
Minimum spanning tree based on MLVA-16 data for *Brucella* strains in China wide

**Fig. 3:**
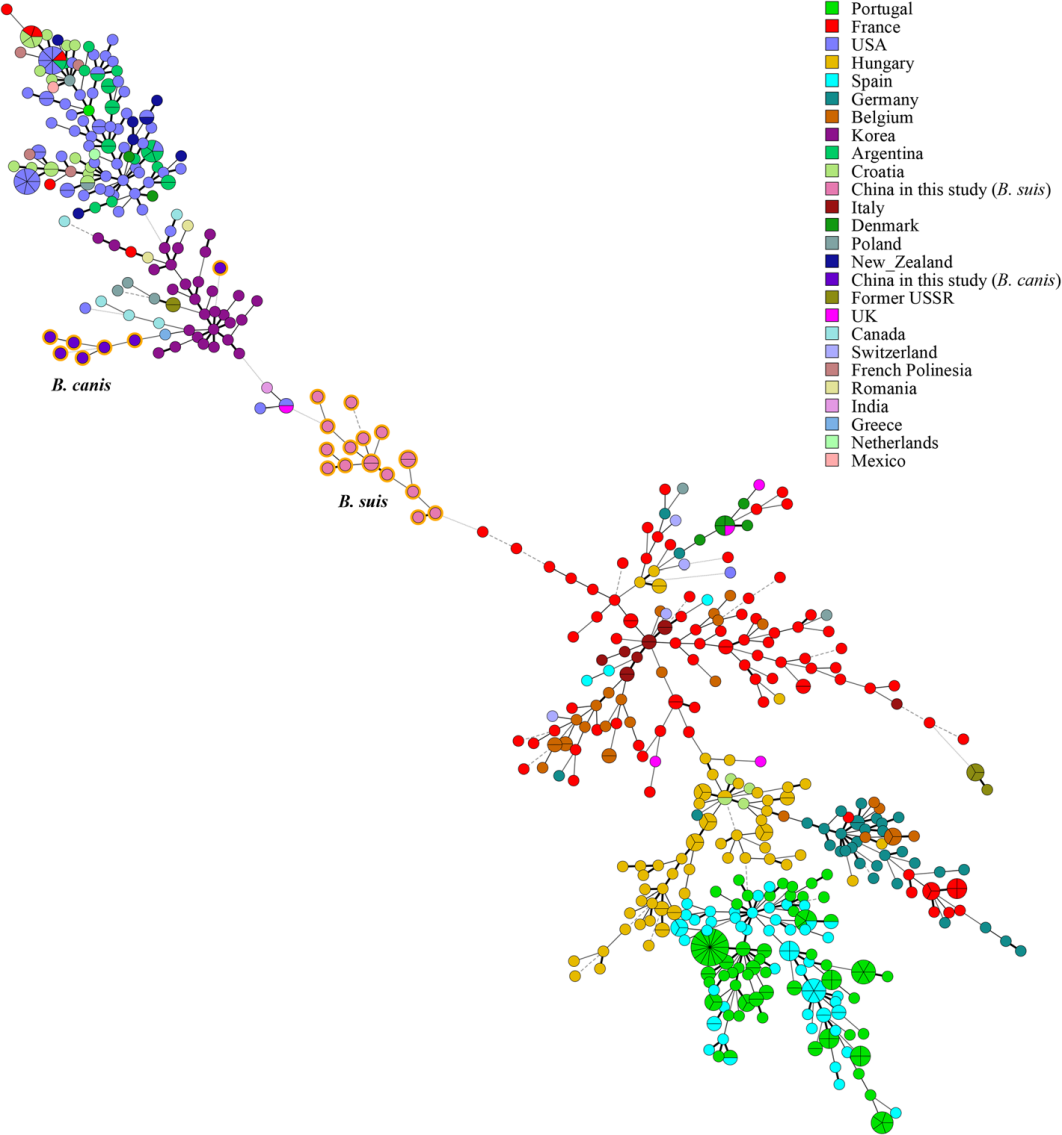
Minimum spanning tree based on MLVA-16 data for *B.* suis and *B. canis* of this study in global level

Based on previously studies, all *B. melitensis* isolates belonged to East Mediterranean lineage, MLVA-11 genotypes 116 represent 81.2% of strains. In contrast, *B. suis* and *B. canis* yielded three novelty MLVA-11 genotypes, respectively; compared with correspond strains from other regions in 2016 MLVAbank. A Minimum Spanning Tree based on MLVA-16 analysis showed that there were five shared genotypes observed among seven *B. melitensis* strains in this study and other regions in China; the remaining nine *B. melitensis* were very close to strains from other provinces. *B. suis and B. canis* from this study represent unique genetic lineage and formed a relative independence branch, which is divergence obviously with strains from China (Fig. 2). In order to determine the origin and assess the genetic relationships of *B. suis and B. canis* from this study, we analyzed MST among strains collected from many parts of the globe. This analysis showed that *B. suis* strains had unique genetic lineages at the global level. However, *B. canis* isolates were very similar to strains from Korea (Fig. 3).

### Cluster analysis for Guangxi Province *Brucella* strains genotypes

A total 34 genotypes were yielded using complete MLVA-16 schedule. Cluster B and C including 6 panel 1 genotypes and comprised by 24 *Brucella* strains were isolated before 2010, and which of four strains were shared two identical MLVA-16 genotypes, respectively; other twenty strains each represent single MLVA-16 genotypes. Cluster A incorporating three panel 1 genotypes and comprised of 15 *Brucella* strains was isolated after 2010. Three MLVA-16 genotypes were shared by two to three strains, and the cluster rate was 44% (7/16). The remaining nine strains each represent single MLVA-16 genotypes.

### Molecular epidemiological investigation of 296 Chinese *B. melitensis* strains

In a representative study, the MLVA-16 schedule was used to investigate molecular relationships between this study *B. melitensis* isolates and 296 *B. melitensis* isolates from other provinces of China. Five shared genotypes were observed in this population (Fig. 2; Additional file 5: Table S5), *B. melitensis* isolates from Guangxi Province had genotypes identical to those of strains from six different provinces, including the Inner Mongolia Autonomous Region, Shanxi, Henan, Shaanxi, Guangdong, and Zhejiang Provinces, and two out of five shared genotypes were comprised by isolates from Guangxi and Inner Mongolia Autonomous Region, 7 out of 16 *B. melitensis* of this province had identical genotypes with strains from six provinces of China. Moreover, GX006 (Fig. 1, GT 26) had identical genotypes with strains from four different provinces, including Shaanxi (2011Jiang#001) and Inner Mongolia (2011Jiang#028, 2013Jiang# 042), Henan (2011Jiang#036), and Guangdong (2011Jiang#027). Moreover, GX008 (Fig. 1, GT 23) obtained from blood samples of patients with brucellosis outbreaks in families in Hezhou City. Subsequently, scene epidemiology found that this family had a history of consuming raw ewe’s milk. This strain had identical genotypes with strains from Inner Mongolia (2013Jiang#045).

### MLST genotyping

**Fig. 4:**
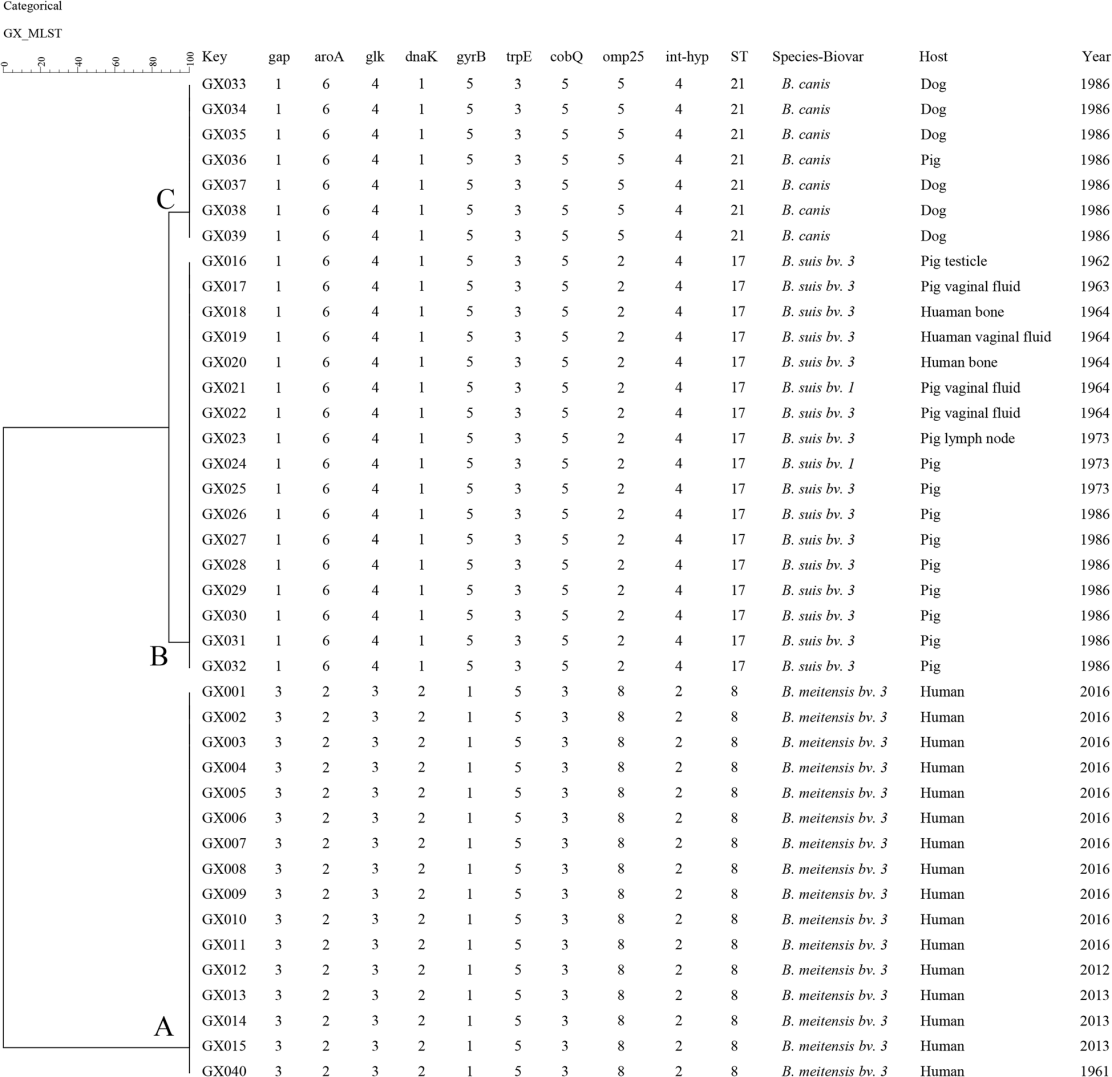
Dendrogram based on the MLST genotyping assay showing relationships of 66 *Brucella* isolates. The columns show the identification numbers, ST types, species-biovar, host, and the year of isolation of the strains

All of isolates were further analyzed by MLST and three known MLST genotypes were identified: ST8 (3–2–3-2-1-5-3-8-2; *n* = 16), ST17 (1–6–4-1-5-3-5-2-4; *n* = 18), ST21 (1–6–4-1-5-3-5-5-4; *n* = 6). Clustering analysis using BioNumerics software showed that the 40 isolates formed three main clusters (A~C) (Fig. 4). All *B. melitensis* were distributed in cluster A and belong to genotype ST8. Cluster B including single genotype ST17 and all strains belonged to *B. suis*; cluster C comprised *B. canis* and belonged to genotype ST21. These strains were divided into three distinct populations based on MLST typing data. There was a visible change in ST of strains isolated from 1961 to 2016 in this province; ST17 predominated among species before the 2000s, but ST8 was the most common population after the 2012. This assay is more suitable for discrimination at species levels and performed population construct investigation of *Brucella* isolates.

## Discussion

In the present study, we employed both MLVA and MLST methods to characterize *Brucella* isolates were obtained from animals and humans in China’s Guangxi Province from 1961 to 2016. Both methods showed an identical testing conclusion that there were at least three *Brucella* species in region examined. Biotyping identified 16 as *B. melitensis* biovar 3, 15 strains as *B. suis* biovar 3 and two as *B. suis* biovar 1, and 7 strains as *B. canis*. Vaccination of breeding pigs with strain S2 has been the main strategy to control *B. suis* brucellosis in this province, but no S2 vaccine strain has been isolated in this study. Our survey showed that a total of 390,630 breeding pigs were vaccinated with strain S2 during 1996–1999, and 600,000 breeding pigs were vaccinated in 2001–2002. The annual average vaccination coverage was higher than 80%, which improved disease control. The prevalence rate of swine brucellosis in this province was 0.31% in 1996 (36/11,521) and 0.00% (0/15,161) in 2004. Based on the time of isolation, *B. suis* biovar 3 was the predominant species in Guangxi before the 2000s, and only three cases of human brucellosis have been reported. Moreover, the circulating species have changed after 2012, with *B. melitensis* biovar 3 becoming the dominant species in this region, resulting in a dramatic increase in human brucellosis. *Brucella* isolates species/biovar typing confirmed that changes in dominant species is one of the main causes of a higher incidence rate of human brucellosis in this region [22]. Subsequently, all of strains were further examined by MLVA-16. Based on panel 1 markers, *B. suis* and *B. canis all* identified three new genotypes. However, none of the *B. suis* or *B. canis* panel 1 genotypes found in our study were identical to any of the genotypes in the *Brucella* 2016 MLVA database. These data indicated that although they had only just been reported, they may have been present historically. This conclusion was consistent with results of etiology analysis. *B. melitensis* was the predominant strain in most provinces, and *B. suis* in Guangxi was the dominant species of *Brucella* [9]. Panel 1 genotype 42 was the most common panel 1 genotype in China, mainly distributed in northeast and northwest of China. Moreover, in present study, 81.2% *B. melitensis* strains were found to belong to panel 1 genotype 42, which was not reported in this area until 2012. This suggested it might be important to explore the molecular epidemiology underlying the higher incidence of human brucellosis in this region after 2012.

All *B. melitensis* strains were from the East Mediterranean group. Genotype 116 was the dominant population and distributed in northwestern China and worldwide in endemic areas of animal and human brucellosis [16], and responsibility for all human brucellosis cases after 2012 in Guangxi province. These data indicated that *B. melitensis* biovar 3 has recently come to circulate in this province. Moreover, Minimum Spanning Tree based on MLVA-16 analysis showed that there were five shared genotypes among *B. melitensis* strains in this study, and strains from other parts of China and the remaining *B. melitensis* were from a single genotype and very similar to strains from other provinces, suggesting that *B. melitensis* in this study may have originated in several different provinces. However, *B. suis* from this study formed a relatively independence branch and represent unique genetic lineage, which visibly diverged from strains from China and other nations; suggestion that epidemic of *B. suis* brucellosis was limited and sporadic cases are predominating. These population has characteristic of historical existence; it was exhibited potential trend for formed independence evolution branch. There was a very close relationship between *B. canis* strains from this study and those from Korea, which is very close to China geographically. The quarantine conditions set for allowing pet animals across national borders are relatively lacking [23].

Complete MLVA-16 locus yielded 34 genotypes and three main clusters (A–C). Cluster A comprised 15 *Brucella* strains were isolated after 2012, there is three MLVA-16 genotypes were shared by two to three strains, and cluster rate was 44% (7/16). This information indicated that 44% (7/16) cases of this region had an outbreak epidemic profile after 2012, and other single genotypes were more sporadic. In addition, GX040 was isolated in 1961 and this strain exhibited unique genotypes when compared to isolates from other provinces in China. This showed that this strain may have been present historically and circulated only within a minor or definite range. Clusters B and C comprised 24 *Brucella* (*B. suis* and *B. canis*) strains isolated before the 2000s, of which four *B. suis* strains had two identical genotypes, suggesting that they had a common source of infection. Another 20 *Brucella* strains each represent single genotypes, indicating that 83.3% (20/24) *Brucella* (*B. suis* and *B. canis*) brucellosis had no epidemiological links or sporadic features, which was also a mainly brucellosis epidemic characteristics in Guangxi before 2012.

In a representative study, molecular epidemiological investigation of 312 Chinese *B. melitensis* strains were performed, showing extensive sharing of genotypes in this population (Fig. 2; Additional file 5: Table S5). *B. melitensis* isolates from Guangxi province had complete identical MLVA-16 genotypes with strains from six different provinces. Previous studies have confirmed that MLVA-16 genotyping results show good correlation with epidemiological data with epidemiologically related isolates displaying identical or very closely related genotypes [24, 25]. These data indicated that these strains may have molecular epidemiological links. Two out of five shared genotypes comprised isolates from Guangxi and the Inner Mongolia Autonomous Region. Seven out of 16 *B. melitensis* of this province had identical MLVA-16 genotypes with strains from five northwest provinces of China, suggesting that these regions were the most likely sources of infections for human brucellosis in Guangxi Province. This conclusion consistent with previous studies [26]. Brucellosis has been spreading from the northern provinces to the south. The proportion of imported brucellosis cases was higher in southern than in northern China after 2000 [11]. Furthermore, GX006 (Fig. 1, GT 26) had identical genotypes with simultaneous strains from four different northern provinces. These data hint that there are cases of cross-infections in these provinces and infected animals may help the contagion spread [27]. Moreover, GX008 (Fig. 1, GT 23) was obtained from blood samples of patients with brucellosis outbreaks in one family in Hezhou City, and this family had a history of consuming raw ewe’s milk from a local farm that had a history of importing sheep northern China. Previous studies proved that consumption raw milk is also an important transmission route to human brucellosis [28]. To our surprised, this strain had identical MLVA-16 genotypes with strains from Inner Mongolia (2013Jiang#045). This suggested that the source of infections for this family of outbreaks was most probably Inner Mongolia. This is consistent with results from an epidemiological survey covering sheep imported from the northern provinces 6 months earlier. Strengthening quarantine inspections of animals crossing borders should become a priority.

MLST analysis revealed that 40 strains were divided into three distinct populations, ST8 (*n* = 16), ST17 (*n* = 18), and ST21 (*n* = 6), respectively. ST17 and ST21 were the main populations in this province before 2000s. This was consistent with previous reports. Guangxi is the southernmost province in China, and it has been a site of brucellosis epidemics caused by *B. suis*. Mostly *B. melitensis* strains belonging to ST8 were found after 2012 and caused a sharp increase in the number of cases of human brucellosis. Moreover, ST8 was commonly distributed between northeastern and northwestern regions in China [21]. Consistent with previous reports, brucellosis caused by *B. melitensis* species has shown a visible trend toward geographic expansion from northern of China to southern provinces [26]. These observations inspired the hypothesis that this province experienced an epidemic of imported human brucellosis caused by *B. melitensis* biovar 3 from northwestern China. Moreover, a total of 3173 serum samples from sheep were assayed during 1996–2003, and none were positive. However, many aborted pregnancies in sheep were observed in this area examined in April 2004, and meanwhile, six positive sheep serum and two positive human serum samples were found. Since then, an epidemiological survey showed that introduction without quarantine from the northwest was the main cause for abortus events in sheep. The rate of human brucellosis was low at 0.35% during 1990–1999, but increased to 7.01% (63/899) in 2004. At present, 624 human brucellosis cases have been reported during 2016–2018; these dates strongly support our hypothesis. However, due to imbalances and patchiness of the number of strains collected among different counties and among different years in this study, in-depth analyses of more *Brucella* strains are required to verify this conclusion and to elucidate the genetic and epidemiological characteristics of *Brucella* species in this province.

## Conclusion

In this work, our team performed a comprehensive investigation of the molecular characteristics of *Brucella* species in Guangxi, China. There were at least three *Brucella* species, *B. melitensis*, *B. suis*, and *B. canis*, in this province. MLVA analysis showed extensive genotype sharing among *B. melitensis* from this study and other provinces within China. The *B. melitensis* isolated in this study may be a recent introduction into this area. *B. suis* formed an independent evolutionary branch and may have spread across a relative limited area. *B. canis* was very similar to strains from Korea. The previously predominant population, ST17, was replaced by the recent emerged ST8 group, which is the predominant ST in most provinces within China. Based on the results of a series of molecular investigations, we hypothesize that Guangxi experienced an imported human brucellosis epidemic caused by *B. melitensis* biovar 3, which might have come from northern China. We consider strengthening of inspections and quarantine of animals and animal products that cross borders imperative to control the imported human brucellosis. Our work provided important clues for enhancing the prevention and control of human and animal brucellosis in this region.

## Supplementary information

**Additional file 1: Table S1.** Table representing strain identification codes (Key), MLVA-8 data, MLVA-11 data, biovars, and year of isolation for 40 *Brucella* isolates.

**Additional file 2: Table S2.** 340 Chinese *Brucella* strains were used to construct the MST (strains in this study are indicated with yellow circles).

**Additional file 3: Table S3.** 615 *Brucella* strains (*B. suis* and *B. canis*) were used to construct the MST on a global scale (strains in this study are indicated with yellow circles).

**Additional file 4: Table S4.** Table representing isolates key, allele characters, ST, species, host, and year of collection of strains were used for MLST analysis.

**Additional file 5: Table S5.** Isolates from Guangxi province had identical MLVA-16 genotypes with strains from other provinces of China.

## Data Availability

All data generated or analyzed during this study are included in this published article and its supplementary information files, will be freely available to any scientist wishing to use them for non-commercial purposes upon request via e-mail with the corresponding author.

## Abbreviations

MLST: Multilocus sequence typing
MLVA: Multiple locus variable-number tandem repeat analysis
PCR: Polymerase chain reaction
ST: Sequence type
